# γ‐Glutamylcysteine Alleviates t‐BHP‐Induced Oxidative Damage in NIH/3T3 Fibroblasts by Promoting Nuclear Translocation of Nrf2

**DOI:** 10.1002/fsn3.71574

**Published:** 2026-02-19

**Authors:** Shuai Lu, Yujie Pan, Mingyan Xia, Jin Luo, Wenfeng Yu

**Affiliations:** ^1^ Department of Biology, School of Basic Medical Science Guizhou Medical University Guiyang China; ^2^ Emergency Department The Affiliated Hospital of Guizhou Medical University Guiyang Guizhou China; ^3^ Department of Anatomy, School of Basic Medicine Science Guizhou Medical University Guiyang China; ^4^ Department of Human Anatomy, School of Basic Medical Science Guizhou Medical University Guiyang Guizhou China; ^5^ Key Laboratory of Human Brain Bank for Functions and Diseases of Department of Education of Guizhou Province Guizhou Medical University Guiyang Guizhou China

**Keywords:** γ‐glutamylcysteine, fibroblast, Nrf2, oxidative stress

## Abstract

Fibroblasts are essential for tissue repair, but reactive oxygen species (ROS) can impair their function, leading to mitochondrial dysfunction and apoptosis. γ‐Glutamylcysteine (γ‐GC), a glutathione (GSH) precursor and potent antioxidant, may protect fibroblasts, though its mechanisms in ROS‐mediated damage remain unclear. This study examined γ‐GC's effects on tert‐butyl hydroperoxide (t‐BHP)‐injured NIH/3T3 fibroblasts. γ‐GC effectively reduced ROS levels, restored antioxidant defenses, and preserved mitochondrial function, thereby inhibiting apoptosis. Mechanistically, γ‐GC upregulated nuclear factor erythroid 2‐related factor 2 (Nrf2) and promoted its nuclear translocation. The Nrf2 inhibitor ML385 confirmed that γ‐GC's protective effects were mediated through Nrf2 activation. These results demonstrate that γ‐GC, as a direct GSH precursor, not only scavenges ROS but also enhances cellular antioxidant capacity and mitochondrial homeostasis. Its dual role in ROS mitigation and Nrf2 activation highlights γ‐GC's therapeutic potential for improving aberrant tissue repair.

## Graphical abstract

γ‐Glutamylcysteine (γ‐GC) reduces reactive oxygen species (ROS) levels in NIH/3T3 fibroblasts via modulation of the intracellular antioxidant system, and mitigates ROS‐induced mitochondrial membrane potential collapse and mitochondrial dysfunction in these cells. γ‐GC further alleviates ROS‐mediated apoptosis in NIH/3T3 fibroblasts. These protective effects of γ‐GC are dependent on its facilitation of Nrf2 nuclear translocation in NIH/3T3 fibroblasts.

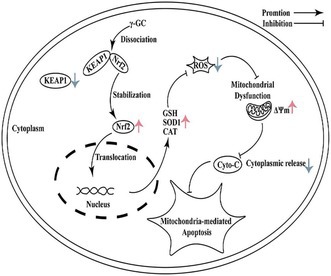

## 1 Introduction

Fibroblasts are present in diverse tissue types and play a crucial role not only in supporting tissue architecture through the deposition and remodeling of the extracellular matrix (ECM), but also in secreting and responding to cytokines, chemokines, and growth factors. Furthermore, they are actively involved in coordinating various biological processes (Davidson et al. 2021). Various forms of tissue damage trigger coordinated interactions among multiple cell types across both temporal and spatial dimensions to restore tissue integrity while minimizing further injury, with fibroblasts playing a pivotal role in this complex process (Correa‐Gallegos et al. 2021). Dysfunction of fibroblasts can lead to impaired tissue healing or the development of fibrosis; thus, focusing on fibroblast function is particularly important for tissue repair.

Oxidative stress is characterized by a disequilibrium between the generation of reactive oxygen species (ROS), such as superoxide anions, hydroperoxides, and hydroxyl radicals, and the intracellular antioxidant defense systems, resulting in cellular dysfunction, damage, and potentially cell death (Bourgonje et al. 2023). An overproduction of ROS can result in abnormalities in fibroblast proliferation and migration (Huang et al. 2020), induce apoptosis (Ren et al. 2022), and cause disruptions in ECM deposition and transformation (Schuster et al. 2023), thereby impeding the tissue repair process. Conversely, when various factors impede the normal tissue repair process, it can lead to chronicity and the overproduction of ROS. This overproduction inhibits fibroblast function, further obstructing tissue repair (Deng et al. 2021). A reduction in intracellular ROS levels may serve as a crucial strategy for enhancing fibroblast function and facilitating tissue repair.

γ‐Glutamylcysteine (γ‐GC), an intermediate in the endogenous synthesis pathway of glutathione (GSH), exhibits numerous biological effects. Research has demonstrated that γ‐GC ameliorates the pathological conditions associated with various diseases, including aging (Lu et al. 2022), degenerative neuropathy (Liu et al. 2021), and inflammatory bowel disease (Zhou et al. 2023). Nonetheless, the potential role of γ‐GC in aberrant tissue repair has yet to be investigated. Thus, this study examined the protective effects of γ‐GC against ROS‐induced damage in fibroblasts and preliminarily elucidated its underlying mechanisms of action, with the objective of informing potential therapeutic strategies to mitigate ROS‐mediated damage during wound healing.

## 2 Materials and Methods

### 2.1 Chemicals and Regents

γ‐GC (CAS No. 636‐58‐8) was obtained from Shanghai Yuanye Bio‐Technology (Shanghai, China). Antibodies against CAT (#21260‐1‐AP, 1:2000), SOD1 (#67480‐1‐Ig, 1:5000), Bax (#60267‐1‐Ig, 1:5000), Bcl‐2 (#68103‐1‐Ig, 1:5000), Cyto‐C (#66264‐1‐Ig, 1:5000), and KEAP1 (#80744‐1‐RR, 1:2000) were purchased from Proteintech (Wuhan, Hubei, China). Antibodies against Caspase‐3 (A17900, 1:1000) and Nrf2 (#A21176, 1:2000) were obtained from ABclonal (Wuhan, Hubei, China). The antibody against Cleaved‐caspase‐3 (#9661, 1:1000) was purchased from Cell Signaling Technology (Danvers, MA, USA). The Cell Counting Kit‐8 (CCK‐8) was acquired from Dojindo Laboratories (Kumamoto, Japan). 4′,6‐diamidino‐2‐phenylindole dihydrochloride (DAPI) was purchased from Invitrogen (Carlsbad, CA, USA). The Cell Viability/Cytotoxicity Assay Kit, GSH and GSSG Assay Kit, ROS Assay Kit, and Mitochondrial Membrane Potential Assay Kit with JC‐1 were obtained from Beyotime (Haimen, Jiangsu, China), while the Annexin V‐FITC/PI Apoptosis Kit was sourced from Lianke (Hangzhou, Zhejiang, China).

### 2.2 Cell Culture

NIH/3T3 fibroblasts were procured from the Shanghai Institute of Biochemistry and Cell Biology (SIBCB). The cells were maintained in Dulbecco's Modified Eagle's Medium (DMEM; Gibco, USA) supplemented with 10% fetal bovine serum (FBS; Gibco, USA) and incubated under a humidified atmosphere at 37°C with 5% CO₂. In all experiments, cells were permitted a 24‐h acclimation period prior to any treatments. Subsequently, the cells were pretreated with or without γ‐GC (20, 40, or 80 μM) for 2 h, followed by the addition of t‐BHP, after which they were cultured for an additional 4 h.

### 2.3 Cell Counting Kit‐8 Assay

The CCK‐8 assay was employed to assess relative cell viability. Briefly, cells were seeded at a density of 5 × 10^3^ cells per well in a 96‐well plate and cultured for 24 h. Cells were pretreated with or without γ‐GC (20, 40, or 80 μM) for 2 h, followed by the addition of t‐BHP, after which they were cultured for an additional 4 h. The culture medium was replaced, and 10 μL of CCK‐8 working solution was added to each well. The plate was then incubated in the dark for 2 h. Subsequently, absorbance at 450 nm was measured using a spectrophotometer.

### 2.4 Western Blot

Cells were pretreated with or without γ‐GC (20, 40, or 80 μM) for 2 h, followed by the addition of t‐BHP, after which they were cultured for an additional 4 h. Cells were collected and lysed using radioimmunoprecipitation (RIPA) lysis buffer supplemented with 1% protease inhibitor. The lysate was then incubated at 4°C for 20 min, during which ultrasonic disruption was intermittently applied. Subsequently, the sample was centrifuged for 20 min at 4°C to obtain the lysate. The protein concentration was determined using a BCA Protein Assay Kit (Thermo Scientific, USA). Protein samples were subjected to electrophoretic separation and subsequently transferred onto a polyvinylidene fluoride (PVDF) membrane. The membrane was then blocked with 5% skim milk at ambient temperature for 2 h. Following this, it was incubated with a specific primary antibody at 4°C overnight and subsequently incubated with a corresponding secondary antibody at room temperature for 2 h. Protein bands were ultimately visualized using a chemiluminescence detection method and analyzed with ImageJ software.

### 2.5 Flow Cytometric Analysis of Apoptosis

NIH/3T3 fibroblasts were cultured in 6‐well plates and incubated for a period of 24 h. Pretreated with or without γ‐GC (80 μM) for 2 h, followed by the addition of t‐BHP, after which they were cultured for an additional 4 h. The cells were digested and collected using Accutase. Approximately 5 × 10^5^ cells were then subjected to treatment with 5 μL of Annexin V‐FITC and 10 μL of PI working solutions, followed by incubation at room temperature for 5 min in the absence of light. The subsequent analysis was conducted using flow cytometry.

### 2.6 Intracellular Glutathione Detection

Cells were pretreated with or without γ‐GC (20, 40, or 80 μM) for 2 h, followed by the addition of t‐BHP, after which they were cultured for an additional 4 h. Cells were collected via centrifugation and protein removal buffers were subsequently added to the pelleted cells, which were then subjected to two rapid freeze–thaw cycles and lysed using liquid nitrogen and a 37°C water bath. The resulting lysate was centrifuged at 4°C at 12,000 *g* for 10 min, and the supernatant was utilized for the quantification of GSH and oxidized glutathione (GSSG).

### 2.7 Reactive Oxygen Species Assay

The ROS Assay Kit employs the fluorescent probe DCFH‐DA, which permeates the cell membrane and is hydrolyzed to yield DCFH, a compound that cannot traverse the cell membrane. Subsequently, intracellular ROS oxidize DCFH, resulting in the formation of the fluorescent compound DCF. In a nutshell, NIH/3T3 fibroblasts were seeded in 6‐well plate for 24 h, pretreatment with or without γ‐GC (80 μM) for 2 h, followed by the addition of t‐BHP, after which they were cultured for an additional 4 h. Then, an appropriate concentration of DCFH‐DA was added to the plate and incubated at 37°C for 20 min in the dark. The fluorescence intensity was measured using flow cytometry and fluorescence microscopy.

### 2.8 Transmission Electron Microscopy

Cells were pretreated with or without γ‐GC (80 μM) for 2 h, followed by the addition of t‐BHP, after which they were cultured for an additional 4 h. Cells were impregnated with 3% glutaraldehyde and subsequently fixed with 1% osmium tetroxide. They were then dehydrated using a gradient series of acetone and embedded in Epon 812 resin. The ultrathin sections were stained with uranium lactate and lead citrate to avoid carbon dioxide deposition. Finally, the sections were examined using transmission electron microscopy (TEM).

### 2.9 Mitochondrial Membrane Potential Assay

The mitochondrial membrane potential (MMP) was evaluated using the JC‐1 probe. When the MMP decreases, JC‐1 remains in its monomeric state, emitting green fluorescence. Conversely, in scenarios of increased MMP, JC‐1 accumulates within the mitochondrial matrix and forms J‐aggregates that exhibit red fluorescence. Pretreat the cells with γ‐GC (80 μM) for 2 h, then add TBHP and continue incubation for 4 h. The cells were stained following JC‐1 staining according to the manufacturer's protocol; visualization was conducted through confocal microscopy and flow cytometry.

### 2.10 Preparation of Cytoplasmic and Nuclear Extracts

The cells were pretreated with γ‐GC (20, 40, or 80 μM) for 2 h, followed by the addition of TBHP and further incubation for 4 h. The cells were washed with PBS and subsequently lysed with cytoplasmic lysate RLN on ice for 20 min, followed by centrifugation at 3000 g for 10 min. The resulting supernatant contained the cytoplasmic protein components. The pellet was then lysed on ice with RIPA lysis buffer for 20 min, with vigorous vortexing every 5 min, and then centrifuged at 12,000 *g* for 20 min. The supernatant obtained from this step contained the nuclear protein components.

### 2.11 Statistical Analysis

Statistical analyses were performed using GraphPad Prism 9. Each experiment was conducted a minimum of three times. The measurement data are expressed as mean ± standard deviation. A one‐way analysis of variance (ANOVA) was employed to determine statistical significance, with a *p*‐value of < 0.05 considered indicative of significance.

## 3 Results

### 3.1 γ‐GC Alleviates t‐BHP‐Induced Apoptosis in NIH/3T3 Fibroblasts

T‐BHP generates a substantial quantity of exogenous superoxide anions, resulting in the depletion of the intracellular antioxidant defense system. This process is utilized to mimic the damage caused by ROS and is extensively employed in research on oxidative stress damage models in vitro (Singh et al. 2019). To investigate the protective effects of γ‐GC on ROS‐induced damage in NIH/3T3 fibroblasts, we exposed the cells to 75 μM t‐BHP for 4 h to establish an in vitro model of oxidative stress injury.

**FIGURE 1:**
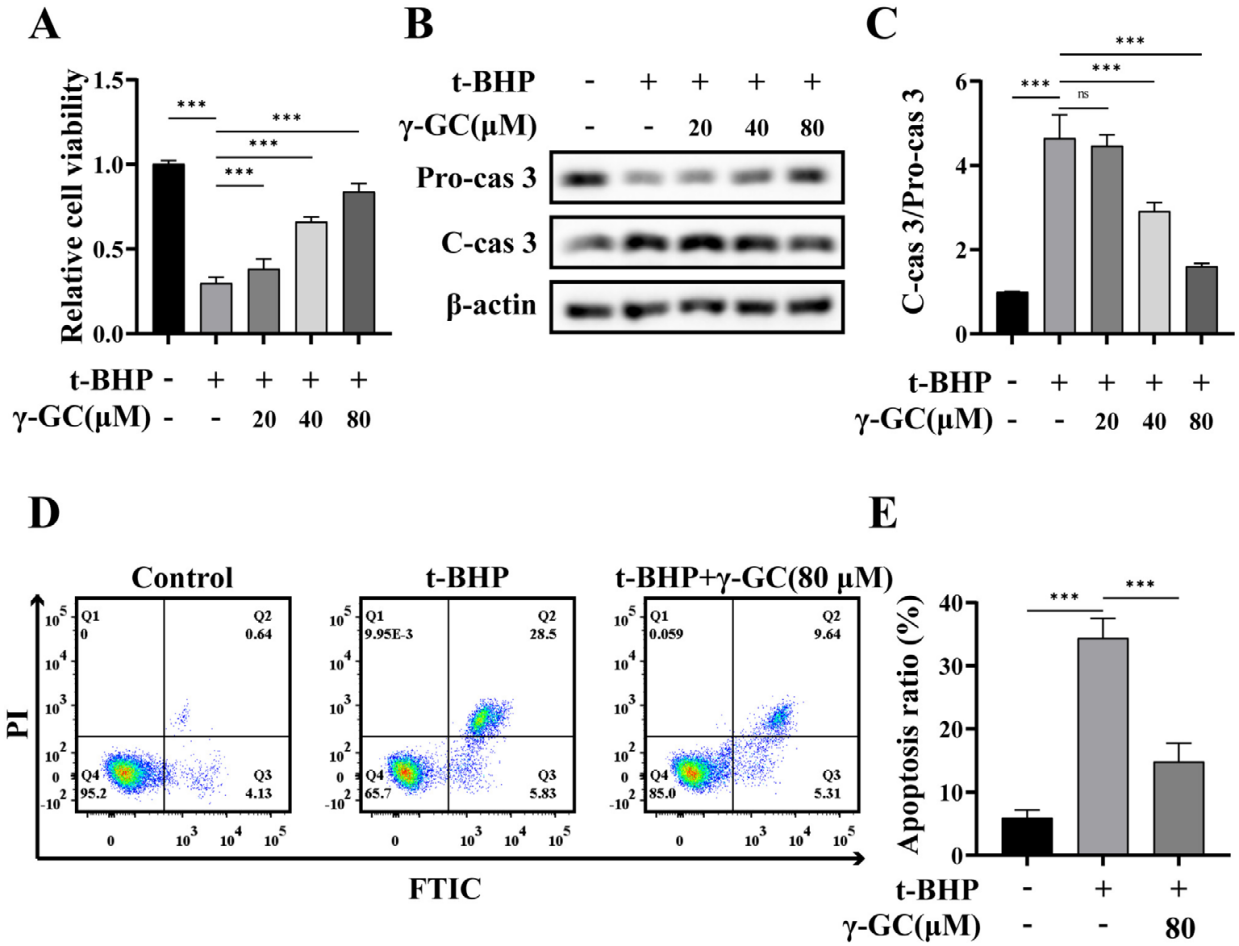
**γ**‐GC alleviates t‐BHP‐induced apoptosis in NIH/3T3 fibroblasts. NIH/3T3 fibroblasts were pretreated with or without γ‐GC (20, 40, or 80 μM) for 2 h, and then stimulated with t‐BHP (75 μM) for 4 h. (A) Cell viability was detected by CCK‐8 assay. (B, C) The protein levels of Pro‐cas 3 and C‐cas 3 were detected by Western blot. NIH/3T3 fibroblasts were pretreated with or without γ‐GC (80 μM) for 2 h, and then stimulated with t‐BHP (75 μM) for 4 h. (D, E) Flow cytometric analysis by Annexin V‐FITC/PI dual staining. The bottom right quadrant represents cells stained with Annexin V‐FITC, indicating early‐stage apoptosis, whereas the top right quadrant signifies cells that are dual‐stained with both PI and Annexin V‐FITC, indicative of late‐stage apoptosis. ***p < 0.001; ns, no significant difference.

As depicted in Figure 1A, cell viability was markedly diminished following t‐BHP treatment; however, γ‐GC demonstrated the capacity to effectively mitigate the t‐BHP‐induced reduction in viability in a dose‐dependent manner. Subsequently, Western blot analysis was employed to assess the protein levels of caspase‐3, revealing that γ‐GC at concentrations of 40 and 80 μM decreased the ratio of cleaved caspase‐3 (C‐cas 3) to pro‐caspase‐3 (Pro‐cas 3), suggesting an inhibitory effect of γ‐GC on t‐BHP‐induced caspase‐3 activation (Figure 1B,C). Furthermore, flow cytometry analysis indicated a substantial increase in Annexin V‐FITC positive cells upon t‐BHP stimulation, whereas treatment with γ‐GC at 80 μM resulted in a significant reduction in Annexin V‐FITC positive cells (Figure 1D,E). These results indicate that γ‐GC protects NIH/3T3 fibroblasts from t‐BHP‐induced apoptosis.

### 3.2 γ‐GC Attenuates t‐BHP‐Induced Oxidative Stress in NIH/3T3 Fibroblasts

**FIGURE 2:**
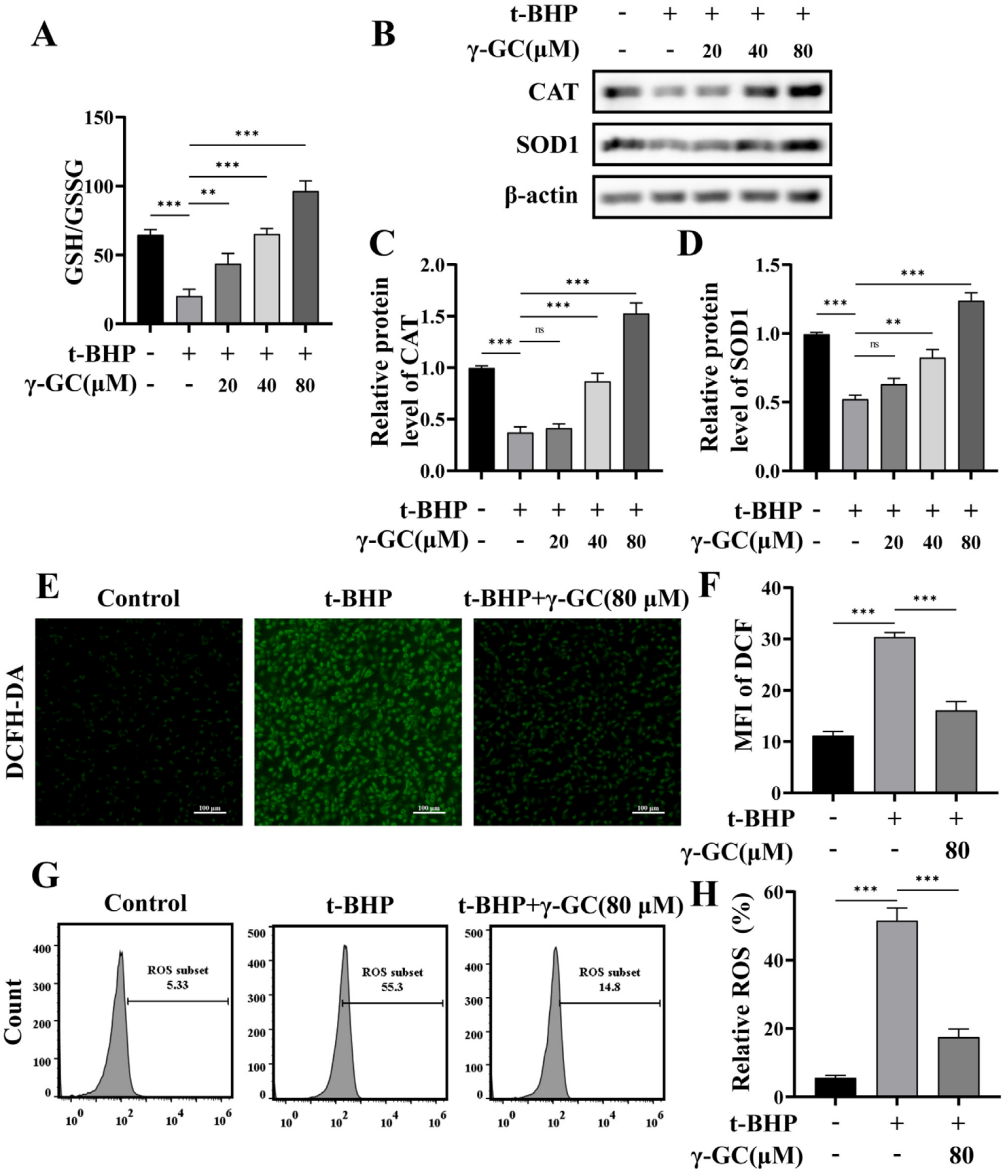
**γ**‐GC attenuates t‐BHP‐induced oxidative stress in NIH/3T3 fibroblasts. NIH/3T3 fibroblasts were pretreated with or without γ‐GC (20, 40, or 80 μM) for 2 h, and then stimulated with t‐BHP (75 μM) for 4 h. (A) The GSH and GSSG assay kit was utilized to quantify the ratio of GSH to GSSG. (B–D) The protein levels of CAT and SOD1 were detected by Western blot. NIH/3T3 fibroblasts were pretreated with or without γ‐GC (80 μM) for 2 h, and then stimulated with t‐BHP (75 μM) for 4 h. (E, F) DCFH‐DA staining and MFI analysis of ROS. Scale bar = 100 μm. (G, H) Flow cytometric analysis of intracellular ROS levels. **p < 0.01; ***p < 0.001; ns, no significant difference.

As a precursor to GSH, γ‐GC can swiftly elevate intracellular GSH levels. Consequently, we measured the concentrations of both GSH and its oxidized form, GSSG, using their ratio as an indicator of antioxidant production levels. Our findings indicated that γ‐GC effectively restored the GSH/GSSG ratio following t‐BHP treatment (Figure 2A). Furthermore, we observed that t‐BHP exposure led to a reduction in the protein levels of superoxide dismutase 1 (SOD1) and catalase (CAT), a trend that γ‐GC (40, 80 μM) reversed in a concentration‐dependent manner (Figure 2B–D). ROS levels were subsequently evaluated using the probe DCFH‐DA, with semiquantitative analysis conducted via mean fluorescence intensity (MFI). The findings indicated an elevation in ROS levels within NIH/3T3 fibroblasts following t‐BHP exposure, which was significantly reduced upon treatment with γ‐GC (Figure 2E,F). Additionally, flow cytometry was employed to assess intracellular ROS levels, yielding consistent results (Figure 2G,H). These findings suggest that γ‐GC enhances the intracellular antioxidant defense mechanisms, thereby attenuating oxidative stress induced by t‐BHP.

### 3.3 γ‐GC Mitigates Mitochondrial Membrane Potential Collapse and Dysfunction Induced by Oxidative Stress in NIH/3T3 Fibroblasts

**FIGURE 3:**
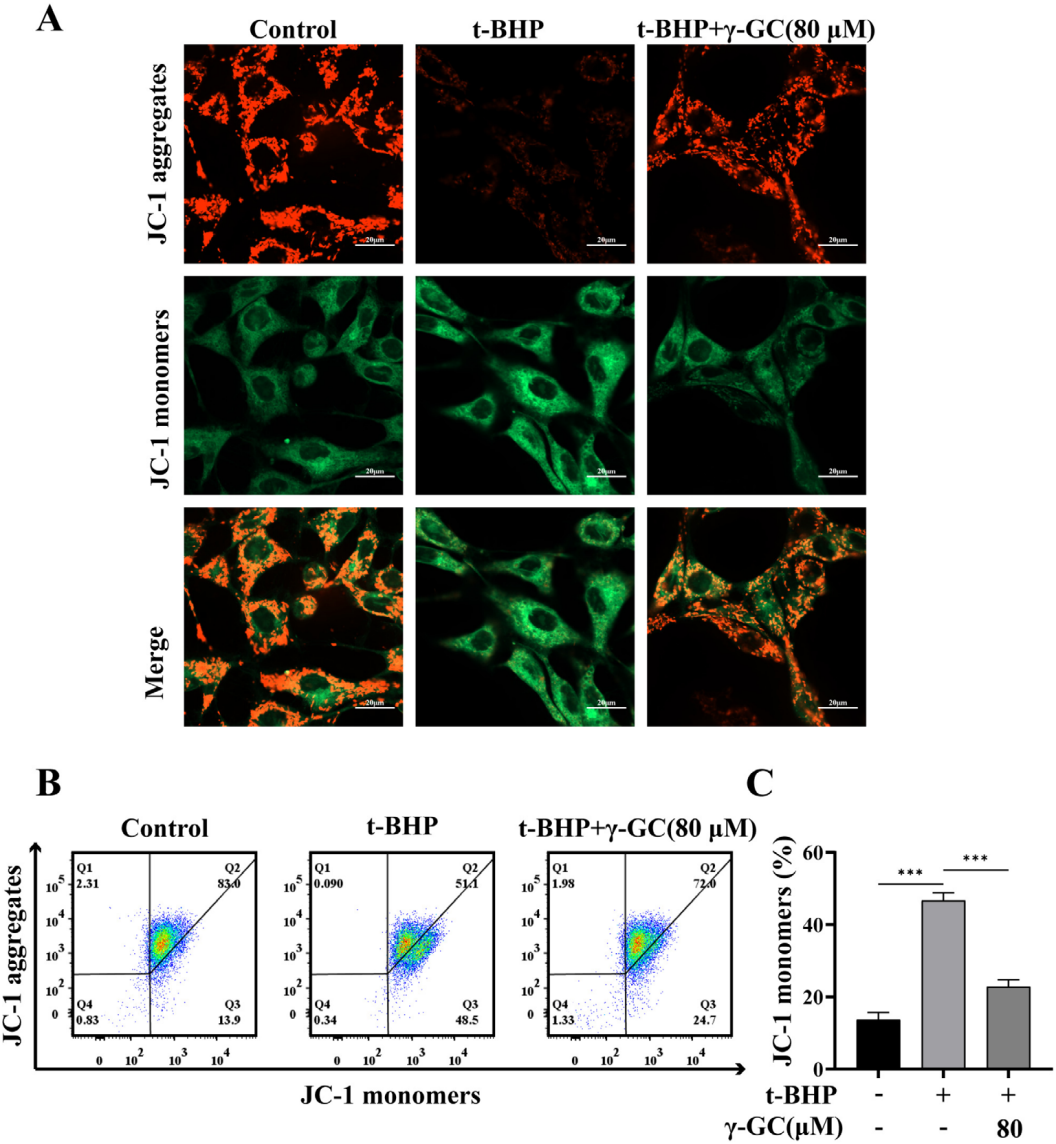
γ‐GC mitigates t‐BHP‐induced mitochondrial membrane potential collapse in NIH/3T3 fibroblasts. NIH/3T3 fibroblasts were incubated with or without γ‐GC (80 μM) for 2 h prior to stimulation with t‐BHP (75 μM) for 4 h. (A) JC‐1 staining was used to visualize MMP. Scale bar = 20 μm. (B, C) Flow cytometry analysis of MMP. ***p < 0.001.

Mitochondria are primary targets for damage induced by ROS, which manifests as the loss of MMP and alterations in mitochondrial morphology (Chen et al. 2018). The impact of γ‐GC on MMP was assessed using the JC‐1 probe, as illustrated in Figure 3A. A marked increase in green fluorescence was observed following t‐BHP exposure, indicating mitochondrial membrane depolarization. In contrast, pre‐treatment with γ‐GC mitigated the t‐BHP‐induced alterations in MMP, as evidenced by a decrease in green fluorescence and a restoration of red fluorescence. These findings were also corroborated by flow cytometry analysis (Figure 3B,C).

**FIGURE 4:**
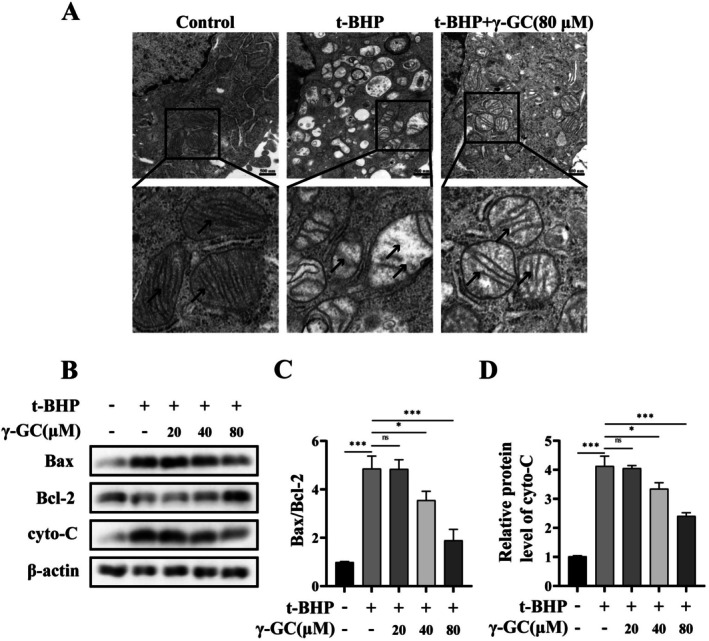
γ‐GC restores t‐BHP‐induced alterations in mitochondrial morphology and dysfunction in NIH/3T3 fibroblasts. NIH/3T3 fibroblasts were incubated with or without γ‐GC (80 μM) for 2 h prior to stimulation with t‐BHP (75 μM) for 4 h. (A) The TEM images of mitochondrial morphology. Scale bar = 500 nm. NIH/3T3 fibroblasts were incubated with or without γ‐GC (20, 40, or 80 μM) for 2 h, followed by stimulation with t‐BHP (75 μM) for 4 h. (B–D) The protein levels of Bax, Bcl‐2, and cyto‐C were detected by Western blot. *p < 0.05; ***p < 0.001; ns, no significant difference.

Transmission electron microscopy analysis revealed that following t‐BHP treatment, there was an increase in mitochondrial volume, a decrease in mitochondrial density, disruption and disappearance of internal cristae, and an indistinct boundary. Conversely, after γ‐GC treatment, an increase in mitochondrial density and restoration of cristae continuity were observed (Figure 4A). The collapse of MMP leads to the release of cytochrome C (cyto‐C) into the cytoplasm and triggers a series of cascade amplification reactions, which ultimately activates caspase 3 and leads to apoptosis (Xue et al. 2023). Western blot analysis was utilized to evaluate the protein levels associated with the mitochondrial pathway of apoptosis (Figure 4B–D). The results demonstrated that stimulation with t‐BHP led to a significant elevation in cyto‐C protein levels, accompanied by an altered Bax/Bcl‐2 ratio. However, pre‐treatment with γ‐GC significantly suppressed the protein levels of cyto‐C and the Bax/Bcl‐2 ratio. These findings indicate that pre‐treatment with γ‐GC attenuates the reduction in MMP and mitigates mitochondrial dysfunction induced by oxidative stress, thereby modulating the apoptotic process.

### 3.4 γ‐GC Promotes the Nuclear Translocation of Nrf2 in NIH/3T3 Fibroblasts

**FIGURE 5:**
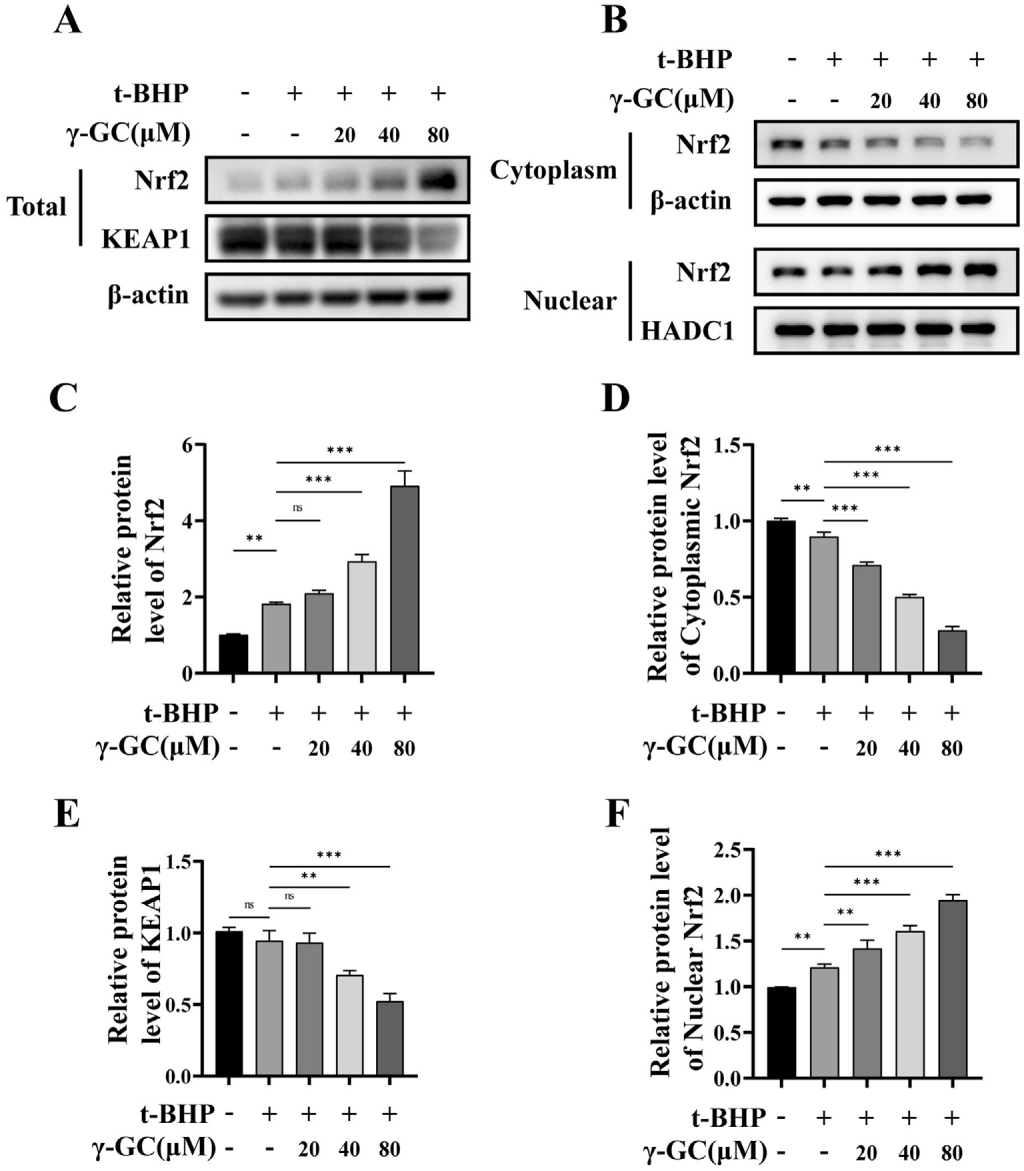
**γ**‐GC promotes the nuclear translocation of Nrf2 in t‐BHP‐induced NIH/3T3 fibroblasts. NIH/3T3 fibroblasts were incubated with or without γ‐GC (20, 40, or 80 μM) for 2 h, followed by stimulation with t‐BHP (75 μM) for 4 h. After this, total protein, as well as the extracted nuclear and cytoplasmic fractions, were subjected to Western blot. (A, C, and E) The total protein levels of Nrf2 and KEAP1. (B, D, and F) The protein levels of Nrf2 in the extracted nuclear and cytoplasmic fractions. **p < 0.01; ***p < 0.001; ns, no significant difference.

The nuclear factor erythroid 2‐related factor 2 (Nrf2) protein levels and subcellular localization are crucial in mediating the antioxidant response (Liu et al. 2022). Our study demonstrated that γ‐GC at concentrations of 40 and 80 μM significantly elevated total Nrf2 protein levels and decreased Kelch‐like ECH‐associated protein 1 (KEAP1) levels, indicating that γ‐GC may exert its biological effects via the Nrf2 signaling pathway (Figure 5A,C,E). Further investigation of the subcellular distribution of Nrf2 revealed that γ‐GC (20, 40, 80 μM) markedly increased the nuclear localization of Nrf2, while concurrently reducing its cytoplasmic levels (Figure 5B,D,F). These results suggest that γ‐GC promotes the nuclear translocation of Nrf2 in NIH/3T3 fibroblasts.

### 3.5 γ‐GC Protects NIH/3T3 Fibroblasts From ROS‐Induced Damage by Promoting Nrf2 Nuclear Translocation

**FIGURE 6:**
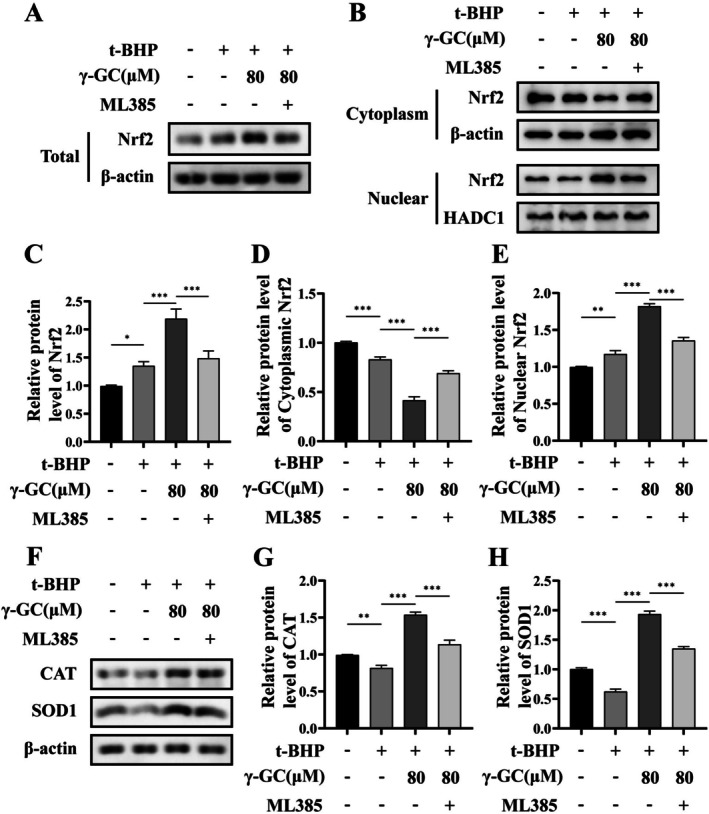
γ‐GC upregulates antioxidant oxidase in t‐BHP‐induced NIH/3T3 fibroblasts by promoting Nrf2 nuclear translocation. NIH/3T3 fibroblasts were pretreated with or without γ‐GC (80 μM) for 2 h, and then t‐BHP (75 μM) and ML385 (2 μM) was added and cultured for 4 h. (A–E) The total Nrf2 protein levels, as well as the levels of Nrf2 protein in the extracted nuclear and cytoplasmic fractions, were assessed using Western blot. (F, G) The protein levels of CAT and SOD1. *p < 0.05; **p < 0.01; ***p < 0.001.

The Nrf2‐specific inhibitor ML385 was employed to further examine the potential of γ‐GC to elicit biological effects by the facilitation of Nrf2 nuclear translocation preservation. As illustrated in Figure 6A–E, treatment with ML385 resulted in a reduction in both the total and nuclear protein levels of Nrf2 compared to the γ‐GC treatment group, while the cytoplasmic protein level of Nrf2 increased. This suggests that ML385 effectively inhibited the promotion of Nrf2 nuclear translocation by γ‐GC. Subsequently, we investigated the downstream protein levels using Western blot. We observed that the inhibition of Nrf2 nuclear translocation counteracted the promoting effect of γ‐GC on the protein levels of SOD1 and CAT (Figure 6F–H).

**FIGURE 7:**
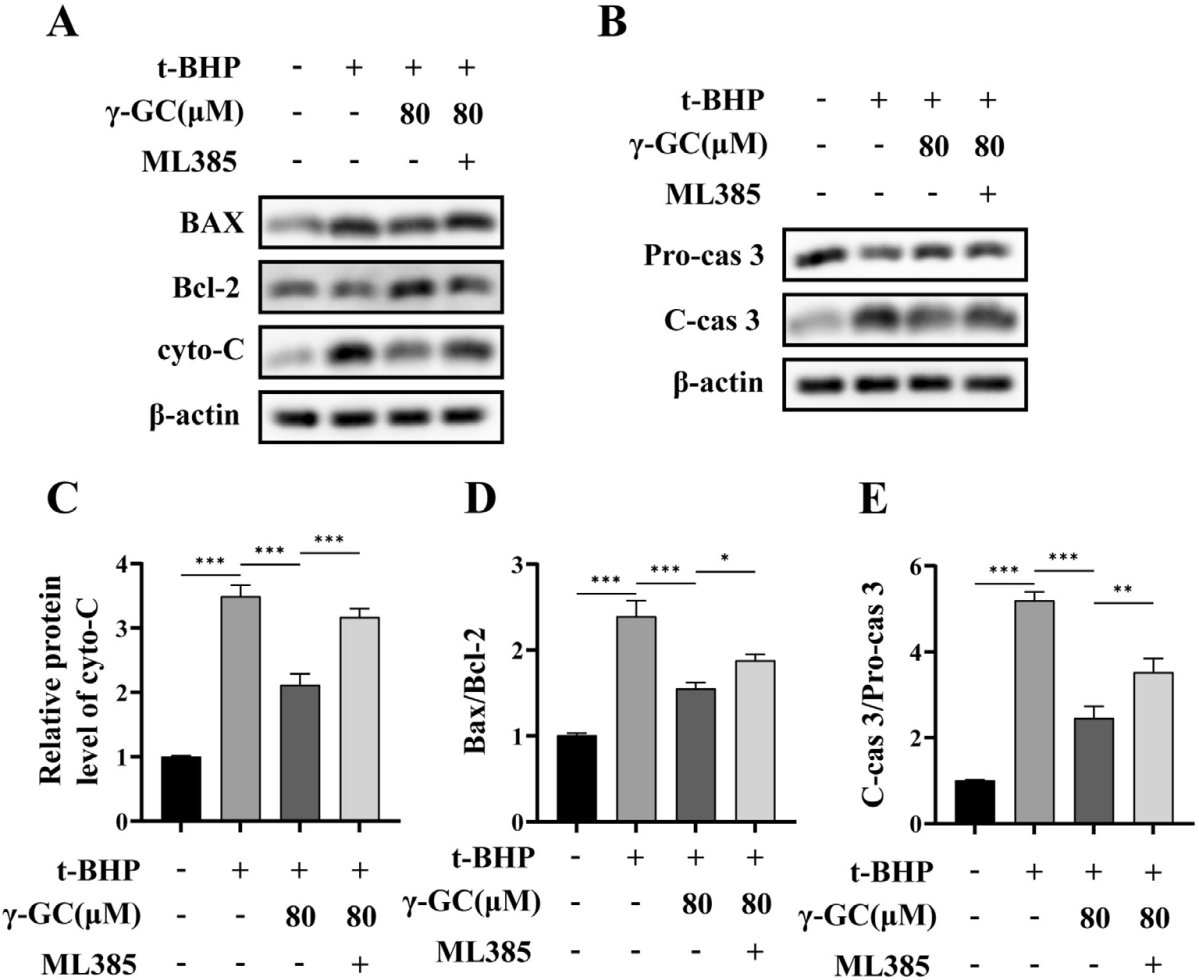
γ‐GC protects NIH/3T3 fibroblasts from ROS‐induced damage by promoting Nrf2 nuclear translocation. NIH/3T3 fibroblasts were pretreated with or without γ‐GC (80 μM) for 2 h, and then t‐BHP (75 μM) and ML385 (2 μM) was added and cultured for 4 h. (A, C, and D) After treatment with ML385, the protein levels of Bax, Bcl‐2, and cyto‐C were assessed using Western blot. (B, E) The protein levels of Pro‐cas 3 and C‐cas 3. *p < 0.05; **p < 0.01; ***p < 0.001.

Furthermore, this inhibition also resulted in the upregulation of apoptosis‐related protein levels associated with the mitochondrial pathway, including cyto‐C, the ratio of Bax to Bcl‐2, and the ratio of C‐cas 3 to Pro‐cas 3 (Figure 7A–E). These findings suggest that γ‐GC contributes to protecting NIH/3T3 fibroblasts from ROS‐induced cellular damage by promoting Nrf2 nuclear translocation.

## 4 Discussion

Fibroblasts, as the key effector cells in tissue repair, play a critical role in driving the repair process through multiple mechanisms. These mechanisms encompass dynamic ECM synthesis and remodeling, such as the secretion of type I/III collagen and regulation of the balance between matrix metalloproteinases and tissue inhibitors of metalloproteinases (Moretti et al. 2022), as well as the maintenance of microenvironmental homeostasis through the secretion of factors like transforming growth factor‐β and vascular endothelial growth factor, which mediate immune‐vascular network interactions (Gauthier et al. 2023). ROS‐induced damage represents one of the most common types of injury to fibroblasts, leading to functional impairment in these cells. Consequently, this can disrupt the tissue repair process and ultimately contribute to the development of related pathological conditions, such as impaired tissue repair or fibrosis. For instance, the accumulation of ROS can induce the senescence of dermal fibroblasts, thereby exacerbating the cellular inflammatory response and leading to the imbalance of ECM deposition (Zhang et al. 2024). In renal fibrotic diseases, ROS exacerbates intracellular oxidative stress by upregulating the activity of enzymes such as NADPH oxidase, which promotes the transformation of fibroblasts into myofibroblasts and accelerates the fibrotic process (Subhash et al. 2024).

The antioxidant defense system constitutes a crucial mechanism for intracellular resistance against ROS and plays a vital role in maintaining redox homeostasis within cells as well as preserving the integrity of cellular functions. This system comprises a diverse array of enzymatic and nonenzymatic antioxidants, including SOD, CAT, glutathione peroxidases, and GSH. These components work in concert to eliminate free radicals and other ROS, thereby protecting cells from oxidative damage (Lan et al. 2023). Nevertheless, excess ROS depletes the antioxidant defense system, resulting in ROS‐induced cellular damage. Furthermore, mitochondria serve as both the primary target and the principal source of ROS‐induced damage. Damage to mitochondria by ROS results in an enhanced release of ROS, thereby establishing a detrimental feedback loop that exacerbates cellular oxidative damage (Glover et al. 2024). Consequently, remodeling the antioxidant defense system and restoring normal mitochondrial function are essential for mitigating the damage caused by ROS.

Numerous studies have demonstrated that γ‐GC, as a precursor of GSH, is transported into cells and catalyzed by glutathione synthetase (GSS) to rapidly increase intracellular GSH level, which demonstrates significant efficacy in augmenting cellular antioxidant capacity (Lu 2009). Specifically, it enhances the activity of antioxidant enzymes and mitigates apoptosis by suppressing cadmium‐induced ROS production, malondialdehyde (MDA) accumulation, and GSH depletion in PC12 cells (Bi et al. 2022). In addition, the anti‐apoptotic potential of γ‐GC has also been confirmed in other non‐fibroblast models, where it can alleviate oxidative stress‐induced apoptosis by regulating the expression of apoptosis‐related signaling molecules (Braidy et al. 2019). In this study, we observed in fibroblasts that γ‐GC could decrease the ratio of C‐cas‐3 to Pro‐cas3, downregulate the Bax/Bcl‐2 ratio, and reduce the number of Annexin V‐FITC‐positive cells. These results are consistent with the conclusions of the aforementioned previous studies, indicating that the anti‐apoptotic properties of γ‐GC are universal across different cell types. Meanwhile, this study further reveals that in fibroblasts, this anti‐apoptotic effect relies on the activation of the antioxidant pathway mediated by Nrf2 nuclear translocation, providing a novel cell‐specific perspective for the research on the anti‐apoptotic mechanism of γ‐GC.

Of greater significance, γ‐GC can function as an antioxidant independently of its conversion to glutathione. However, there are few studies on the interactions between γ‐GC and mitochondria. In this study, t‐BHP‐induced NIH/3T3 fibroblasts served as a ROS injury model to explore the antioxidant mechanisms of γ‐GC in vitro. The findings indicate that treatment with γ‐GC led to an increase in intracellular antioxidants, including GSH, CAT, and SOD1, thereby restructuring the antioxidant defense system. It is noteworthy that our observations indicate an increase in the GSH/GSSG ratio at a concentration of 20 μM for γ‐GC; however, there was no corresponding increase in CAT and SOD1 levels, nor were there subsequent anti‐apoptotic effects. These findings suggest that the antioxidant efficacy of γ‐GC may be concentration‐dependent. Additionally, we found that γ‐GC can inhibit apoptosis by restoring the MMP and repairing the integrity of mitochondrial cristae structure in t‐BHP‐injured fibroblasts. This result echoes the mitochondrial protective effect of γ‐GC revealed in the study by Jennifer Drake et al. (Drake et al. 2003), confirming that the role of γ‐GC in regulating mitochondrial function is stable across different cell types. Meanwhile, providing key mitochondrial‐level mechanistic support for the application of γ‐GC in tissue repair scenarios.

Nrf2, functioning as a transcription factor that modulates cellular oxidative stress, is capable of initiating the transcription of antioxidant‐related genes. The classical regulation of Nrf2 involves its interaction with KEAP1. In the resting state, Nrf2 is bound by KEAP1, which anchors it in the cytoplasm and inhibits its activity by promoting ubiquitination and subsequent degradation (Ngo et al. 2022). Upon dissociation from KEAP1, Nrf2 translocates to the nucleus, where it binds to the ARE or the electrophilic response element (EpRE) of target genes. This binding activates the expression of downstream genes, such as *GSH*, *CAT*, and *SOD* (Raghunath et al. 2018). Nrf2 is essential for the tissue repair process, and its inhibition leads to ongoing oxidative stress and inflammation, delaying healing (He et al. 2022; Li et al. 2019). Previous studies on neural tissues and cells have shown that γ‐GC can alleviate oxidative stress damage and inflammatory responses by activating the Nrf2 pathway, thereby improving the pathological processes associated with neurodegenerative diseases (Zhang et al. 2022). Different from the findings in neural cells, this study observed in fibroblasts that γ‐GC not only upregulates the total protein level of Nrf2 but also significantly promotes Nrf2 nuclear translocation by downregulating KEAP1 expression. This process is a crucial prerequisite for γ‐GC to enhance the expression of antioxidant enzymes (SOD1, CAT) and inhibit the mitochondrial apoptotic pathway in fibroblasts. In addition, the activation of Nrf2 by γ‐GC in neural cells mainly serves the purpose of neuroprotection, whereas the Nrf2 activation mediated by γ‐GC in this study is more directly related to the core function of tissue repair—specifically, the physiological processes involved in maintaining fibroblast viability and promoting ECM synthesis.

In investigating the antioxidant mechanism of γ‐GC, it has been observed that this compound not only elevates the total protein levels of Nrf2 but also facilitates its nuclear translocation, thereby initiating the transcription of downstream genes associated with antioxidant activity. Notably, at a γ‐GC concentration of 20 μM, there was no significant increase in total Nrf2 protein levels; however, an increase in nuclear Nrf2 protein levels was observed, accompanied by a decrease in cytoplasmic Nrf2 levels. This suggests that γ‐GC plays a crucial role in enhancing the nuclear translocation of Nrf2.

In summary, fibroblasts are integral to tissue repair and regeneration; however, the overproduction of ROS can impede this process. Consequently, the remarkable capacity of γ‐GC to mitigate ROS levels in fibroblasts makes it a promising candidate for the treatment of abnormal tissue repair.

## 5 Conclusion

γ‐GC attenuated ROS levels in t‐BHP‐induced NIH/3T3 fibroblasts, restored the antioxidant defense system, reinstated normal mitochondrial function, and inhibited the ROS‐induced mitochondrial apoptotic pathway. Moreover, γ‐GC confers a protective effect against ROS‐induced damage in fibroblasts by promoting Nrf2 nuclear translocation.

## Author Contributions

Shuai Lu: methodology, software, writing – original draft. Yujie Pan: methodology, writing – review and editing. Mingyan Xia and Jin Luo: software, methodology. Wenfeng Yu: funding acquisition, supervision.

## Funding

This work was supported by the Science and Technology Fund Project of Guizhou Provincial Health Commission (Grant no.: gzwkj2024‐274), Guizhou Provincial Science and Technology Projects (ZK [2024] General 146), and the Guizhou Provincial Health Commission (Grant no.: gzwkj2024‐397).

## Conflicts of Interest

The authors declare no conflicts of interest.

## Data Availability

The data that support the findings of this study are available on request from the corresponding author. The data are not publicly available due to privacy or ethical restrictions.
